# Osteosarcoma and Metastasis Associated Bone Degradation—A Tale of Osteoclast and Malignant Cell Cooperativity

**DOI:** 10.3390/ijms22136865

**Published:** 2021-06-25

**Authors:** Kirstine Sandal Nørregaard, Henrik Jessen Jürgensen, Henrik Gårdsvoll, Lars Henning Engelholm, Niels Behrendt, Kent Søe

**Affiliations:** 1 Finsen Laboratory, Rigshospitalet/Biotech Research and Innovation Center (BRIC), University of Copenhagen, 2200 Copenhagen, Denmark; hjj@finsenlab.dk (H.J.J.); gvoll@finsenlab.dk (H.G.); lhe@finsenlab.dk (L.H.E.); niels.behrendt@finsenlab.dk (N.B.); 2 Clinical Cell Biology, Pathology Research Unit, Department of Clinical Research, University of Southern Denmark, 5230 Odense, Denmark; kent.soee@rsyd.dk; 3 Clinical Cell Biology, Department of Pathology, Odense University Hospital, 5000 Odense, Denmark; 4 Department of Molecular Medicine, University of Southern Denmark, 5230 Odense, Denmark

**Keywords:** bone degradation, osteosarcoma, bone metastasis, vicious cycle, osteoclast

## Abstract

Cancer-induced bone degradation is part of the pathological process associated with both primary bone cancers, such as osteosarcoma, and bone metastases originating from, e.g., breast, prostate, and colon carcinomas. Typically, this includes a cancer-dependent hijacking of processes also occurring during physiological bone remodeling, including osteoclast-mediated disruption of the inorganic bone component and collagenolysis. Extensive research has revealed the significance of osteoclast-mediated bone resorption throughout the course of disease for both primary and secondary bone cancer. Nevertheless, cancer cells representing both primary bone cancer and bone metastasis have also been implicated directly in bone degradation. We will present and discuss observations on the contribution of osteoclasts and cancer cells in cancer-associated bone degradation and reciprocal modulatory actions between these cells. The focus of this review is osteosarcoma, but we will also include relevant observations from studies of bone metastasis. Additionally, we propose a model for cancer-associated bone degradation that involves a collaboration between osteoclasts and cancer cells and in which both cell types may directly participate in the degradation process.

## 1. Introduction

Bone cancer can occur due to the development of primary bone tumors or as the result of metastasis formation by disseminated cancer cells from distant organs. Osteosarcoma is the most prevalent type of primary malignancy in bone. The incidence of osteosarcoma is bimodal with the first peak occurring during the growth spurt in late childhood/adolescence and a second peak in the elderly population [1,2]. For secondary cancers, bone is the third most common site for metastasis in general, but with considerable variation between cancer types. It is most prominent in patients with prostate cancer, of which 30% develop bone metastases within 10 years after diagnosis of the primary tumor, followed by 13% of lung cancer patients, 10% of renal cancer patients, 8% of breast cancer patients, and 3% of colorectal and malignant melanoma patients [3]. The incidence is dramatically increased, if you only consider patients with advanced (stage IV) cancer. Notably in prostate and breast cancer, the incidence is increased to 70 and 60%, respectively, in this patient group [3].

Both osteosarcoma and bone metastases are highly devastating for patients. For osteosarcoma patients with localized disease, the 5-year overall survival is 50–70%. However, the 5-year overall survival is less than 30% for patients who either present with metastasis at the time of diagnosis, develop metastasis during disease progression, or show a poor response to chemotherapy [4,5,6]. The prognosis of patients with bone metastases originating from a primary cancer in the soft organs is also often poor. Based on data from a Danish cohort study, Svensson and colleagues reported that in the period from 1994 to 2012 the 5-year survival rate after diagnosis of bone metastasis was only 1% for lung cancer, 3% for colon cancer, 5% for kidney and melanoma, 6% for prostate cancer, and 13% for breast cancer [7]. These numbers are supported by other reports covering the same period for Danish and international cohorts [8,9,10,11,12,13]. Survival rates are likely to have improved since then [14,15,16], but bone metastasis remains an incurable condition. Contributing to the poor prognosis are the consequences of bone metastases such as anemia, increased susceptibility to infection, life-threatening hypercalcemia, spinal cord compression, severe pain, fatigue, increased risk of skeletal fractures, and decreased mobility [17,18,19,20].

Remodeling and resorption of bone play a key role in the establishment and progression of bone cancer. Consequently, an understanding of the mechanisms of bone turnover in the various types of bone cancer is of central importance. Several cell types and proteins are involved in this process, and it is beyond the scope of the review to include a detailed description of them all. Here, we will focus on the role of the osteoclast as a driver of cancer-associated bone degradation and its significance throughout the course of disease. We will also discuss indications of direct bone-degradation actions mediated by the malignant cells and propose a model that encompasses a cooperation between osteoclasts and cancer cells, which is distinct from that of the vicious cycle model.

## 2. Physiological Bone Remodeling

Continuous remodeling of bone is a crucial process for maintaining healthy bone throughout life. Besides the obvious biomechanical properties of the skeleton, bone is also a reservoir of minerals and growth factors. Furthermore, bone creates the spatial environment of the bone marrow, which is, e.g., the primary site for hematopoiesis [21,22,23]. Bone health is therefore an integral part of the general health status.

Bone remodeling consists of bone resorption by osteoclasts and new bone formation by osteoblasts, with these two events being linked through the reversal phase and reversal cells. For a thorough outline, physiological bone remodeling is reviewed in [24], and the significance and biological properties of reversal cells were recently reviewed in detail in [25]. Here, we will briefly introduce some important concepts in physiological bone remodeling that are of relevance for this review.

During bone resorption, osteoclasts attach to bone, become polarized, form a sealing zone, and release protons into this extracellular compartment. This leads to disintegration of the hydroxyapatite crystals, and exposure of the organic bone component, which mainly consists of interlinked collagen type I fibrils and fibers. The degradation of this material is also facilitated by the acidic microenvironment, which creates ideal conditions for collagen-degrading cysteine proteases, in particular cathepsin K [26,27,28]. In the subsequent bone formation phase, osteoblasts synthesize and deposit organic bone matrix (osteoid), that is mineralized by hydroxyapatite crystals [24]. In addition to osteoclasts and osteoblasts, reversal cells are important to ensure bone homeostasis under physiological conditions. Reversal cells are osteoprogenitors that are found on osteoclast-eroded bone surfaces. Reversal cells next to osteoclasts have been implicated in both supporting osteoclast-mediated resorption and in the removal of collagen debris on the eroded bone surfaces by means of collagenase activity. Both activities prepare the eroded bone surface for subsequent bone formation. Interestingly, linking bone resorption and formation, observations in the literature indicate that reversal cells differentiate into bone forming osteoblasts (recently reviewed in detail in [25]).

The necessary coordination of bone resorption and formation is ensured by mechanisms that provide positive or negative regulation of both osteoclastic bone resorption and osteoblastic bone formation. Especially negative regulation of bone resorption and stimulation of bone formation is important, because osteoclastic bone resorption is a much faster process (weeks) than osteoblastic bone formation (months) [29]. Important regulators of osteoclastic bone resorption activity are: (1) the receptor for activation of nuclear factor kappaB (RANK), (2) RANK ligand (RANKL), and (3) osteoprotegerin (OPG). Both RANKL and OPG are expressed by osteoblast-lineage cells; osteocytes embedded within the bone matrix [30,31], bone lining cells [32], bone remodeling compartment canopy cells [33], and reversal cells [33]. RANKL will facilitate both the formation of osteoclasts and boost their resorptive activity, while OPG (a soluble decoy receptor for RANKL) is secreted to ensure that osteoclast formation and activity does not get out of control. Furthermore, the expression of macrophage colony-stimulating factor (M-CSF), also by osteoblast-lineage cells, is a prerequisite for osteoclastic progenitors to express RANK and thereby priming these precursors to be able to respond to RANKL. The proper management of these three regulators by osteoblast-lineage cells is important to ensure that osteoclastic activity is kept in check. In this context, it is important to bear in mind that the expression of these three factors is not only regulated in time, but also in space [34]. Osteoclastic bone resorptive activity is on the other hand also a key facilitator to boost bone formation. An illustrative example of this is that during bone resorption osteoclasts release growth factors that are imbedded in the bone matrix and which are thought to facilitate the generation of osteoblast progenitor cells and their differentiation into bone forming osteoblasts. For a thorough presentation on this process, please refer to the recent review in [35].

## 3. The Vicious Cycle of Cancer-Mediated Bone Degradation

In both primary bone cancer and bone metastases, the bone remodeling process creates a favorable environment for tumor establishment and progression. For a tumor to expand, important needs include growth factors to support proliferation and survival, but also space to allow the cancer cells to expand. The “vicious cycle” of cancer-associated bone degradation represents a manipulated version of the physiological bone remodeling process, which fulfills these criteria. This mechanism was initially identified in bone metastasis and has been intensely studied for decades [36,37,38,39,40,41,42]. The mechanisms of bone degradation in osteosarcoma have not been studied as extensively as it has for bone metastasis. Perhaps this is due to the low incidence of osteosarcoma; osteosarcoma makes up less than 1% of all cancer diagnoses [43]. However, the same processes have also been suggested to play a role in osteosarcoma [44,45,46].

In the vicious cycle model, tumor cells will stimulate an exaggerated formation and activation of osteoclasts by directly or indirectly facilitating the local production of cytokines and growth factors, including interleukin (IL)-1, IL-6, IL-8, IL-11, prostaglanding E3, tumor necrosis factor alpha (TNF-α), M-CSF, vascular endothelial growth factor (VEGF), and RANKL [44,46,47,48,49,50,51,52,53,54,55]. Through the secretion of, e.g., parathyroid hormone-related protein (PTHrP), the tumor cells can also stimulate expression of RANKL and reduce expression of OPG by osteoblast-lineage cells [38,56]. The over-stimulated osteoclastogenesis leads to exaggerated bone resorption and ultimately to pathological bone destruction. Some of the fuel for the vicious cycle comes through the release of bone-embedded growth factors. Under physiological conditions, these growth factors facilitate the generation of osteoblast progenitors [35], but when cancer cells are present, they will also stimulate tumor cell growth. Examples of these growth factors include transforming growth factor beta (TGF-β), insulin-like growth factors (IGFs), fibroblast growth factors (FGFs), bone morphogenic proteins (BMPs), and platelet derived growth factor (PDGF) [56,57]. In addition, cancer cells express the calcium-sensing receptor, which allows the calcium released during bone resorption to further promote cancer cell proliferation and PTHrP release [41,58,59,60,61,62].

## 4. The Role of Osteoclasts in Bone Cancer Progression and Cancer-Mediated Bone Destruction

**Figure 1:**
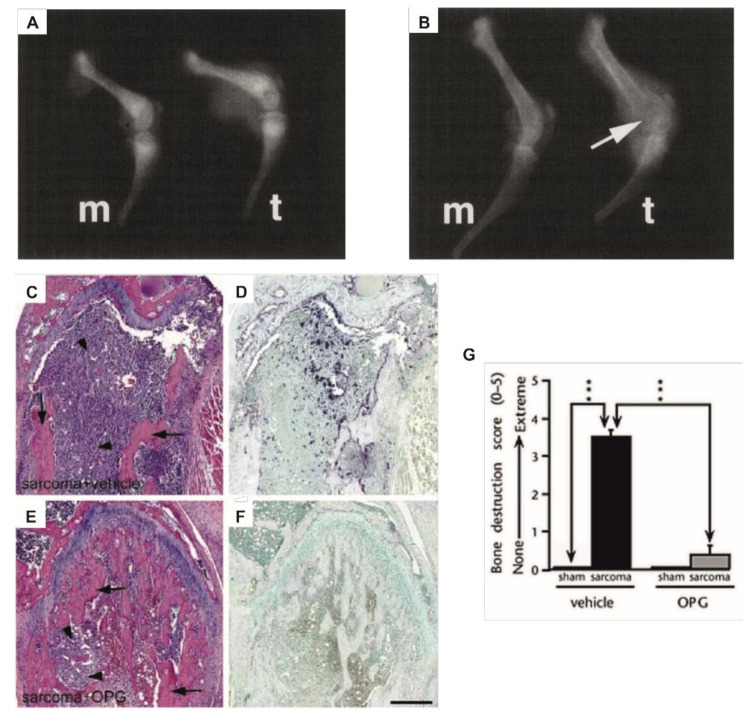
Examples of a central role of osteoclasts in primary bone cancer-induced osteolysis. (**A**,**B**) X-ray images of femurs from (**A**) osteoclast-deficient strain B6C3Fe-a/a-Mitf^mi^ or (**B**) wild type mice, 14 days after inoculation with medium alone (m) or osteolytic sarcoma cells (t). Osteolysis (arrow) was only observed in the wild type mice. (**C**–**F**) Sections of sarcoma-inoculated mouse femurs stained with hematoxylin and eosin (**C**,**E**) or tartrate-resistant acid phosphatase, staining osteoclasts dark violet (**D**,**F**). Arrows indicate bone and arrow heads indicate tumor cells. OPG treated mice (**E**,**F**) showed greatly reduced bone destruction and diminished osteoclast numbers compared to vehicle treated mice (**C**,**D**). (**G**) Bone destruction score in sham and sarcoma inoculated mice from the same experiment as **C**–**F**, treated with OPG or vehicle. Data are shown as mean (± s.e.m.). *** *p* < 0.001, one-way ANOVA, Fisher PLDS, arrows indicate the groups being compared. (**A**,**B**) Reproduced with kind permission from Clohisy and Ramnaraine, Journal of Orthopaedic Research; published by John Wiley and Sons, 1998 [63]. (**C**–**G**) Reproduced with kind permission from Honore et al., Nature Medicine; published by Springer Nature, 2000 [64].

Osteosarcoma arises from transformed pre-osteoblasts and osteoblasts and is defined by the production of osteoid. Yet, in most cases of osteosarcoma, the tumors are also osteolytic [6]. As in physiological bone development and homeostasis, the osteoclast is considered the main player when it comes to bone degradation in primary bone cancer, and it plays an intricate role in tumor progression. This was demonstrated in a study where sarcoma cells with an osteolytic potential were inoculated into the femur of osteoclast-deficient mice. In this case, no tumor-induced bone destruction was observed (Figure 1A), and tumor size was greatly reduced in comparison with osteoclast-sufficient host mice (Figure 1B) [63].

Osteoclastogenesis depends on RANK-RANKL signaling and therefore a deletion of RANK expression in myeloid lineage cells results in a lack of osteoclast precursors and mature osteoclasts [65]. In combination with a genetic mouse model, MOTO, that spontaneously develops aggressive osteosarcoma with lung metastasis [66], the elimination of osteoclasts resulted in delayed tumor onset, reduced number of metastatic lesions in the lungs, and prolonged survival [65]. Evaluation of tumor growth in the tibia of RANKL-deficient mice revealed a decrease in growth rates, that was most prominent 10 days after injection of osteosarcoma cells, and these mice also developed fewer metastatic lesions in the lungs in comparison with wild type mice [67]. When mice with osteolytic sarcoma or osteosarcoma were treated with OPG or RANK-Fc (a recombinant RANKL antagonist), resulting in a decrease in osteoclast numbers, protection against tumor-induced lytic bone lesions was observed (Figure 1C–G) [45,64,68]. Furthermore, although the anti-resorptive therapy had no direct effect on the cancer cells, treatment of the host mice with OPG or RANK-Fc resulted in reduced tumor growth and increased survival [45,64,68]. This underscores the importance of the bone resorbing osteoclasts in the progression of primary bone cancer.

In bone metastasis, bone resorbing osteoclasts have also been identified as the cell responsible for the cancer-induced bone degradation. Active bone resorbing osteoclasts are observed in areas with tumor-induced osteolysis, and compared to normal bone, the number of osteoclasts is increased, as is the osteoclast size, and number of nuclei in the osteoclasts [51,53,69,70,71,72,73,74,75]. In mice, with nonfunctioning osteoclasts due to genetic deficiency of *src* or *β*_3_ integrin, bone metastatic melanoma cells fail to induce destruction of bone [76]. Osteoclastogenesis can be inhibited by a monoclonal antibody against PTHrP [38]. In a mouse model of experimental osteolytic breast cancer metastasis, cancer-induced bone degradation was inhibited and a dramatic reduction in bone tumor growth was observed in the PTHrP-antibody-treated mice [38]. Additionally, a genetic deficiency of the transmembrane receptor CXCR4 in hematopoietic cells, which leads to increased osteoclastogenesis, number of nuclei per osteoclast, and bone resorptive activity, results in larger bone metastases with higher growth rates, after injection of melanoma cells into the left cardiac ventricle. This increase in bone tumor burden is not accompanied by an increased tumor burden in other organs [77].

Altogether, the studies described in this section emphasize a key role of osteoclast-mediated osteolysis throughout the course of disease in both primary and secondary bone cancer.

## 5. Involvement of Tumor Cells in Bone Degradation

Although numerous studies have established the osteoclast as the main actor responsible for the bone degradation processes, as discussed above, there are also observations, which imply a more direct role of the malignant cells in cancer-mediated bone degradation. We have found sarcoma cells cultured in vitro on devitalized bovine bone slices to be capable of degrading the exposed bone collagen [70]. These sarcoma cells express both the collagenolytic protease membrane type 1-matrix metalloproteinase (MT1-MMP/MMP14) and the endocytic collagen receptor, urokinase plasminogen activator receptor-associated protein (uPARAP/Endo180), two membrane-associated proteins with established roles in bone remodeling [78]. Blocking MMPs and uPARAP/Endo180 had a strong inhibitory effect on the uptake of collagen for intracellular degradation in the lysosomes by the sarcoma cells [70].

Similarly, breast cancer [79,80], prostate cancer [81], melanoma [82], and myeloma [83] cells have also been implicated in osteoclast-independent bone degradation in vitro. Direct cancer-cell mediated bone resorption was investigated by culturing cell lines, representing the mentioned cancer types, on devitalized fetal rodent long bone [79,83], calvariae [81,82], or on an osteoid-like matrix deposited by osteoblastic osteosarcoma cells [80,81,82]. Bone resorption was evaluated by an increased release of incorporated 3H-proline [79,80,81,82], reflecting collagenolysis, or 45Ca [79,81,82,83], reflecting demineralization. As for osteolytic sarcoma, MMP inhibitors blocked cancer-cell mediated osteolysis, suggesting a role of cancer cell-expressed MMPs in the collagenolytic process [80,81,82]. Additionally, indications of resorption cavities were found after culture of breast cancer, prostate cancer, and melanoma cells on devitalized bovine bone slices [80,81,82]. However, the characteristics of these resorption cavities were not compared to resorption cavities generated by osteoclasts, and therefore the efficiency of this process is uncertain.

Indications for a direct involvement of tumor cells in the degradation process have also been obtained through in vivo studies, as discussed below. However, when comparing the numerous studies in mice, the use of different cancer cell lines or mouse models is likely to contribute to the complicated pattern that emerges from the published literature. Some of these models may have unique features that will influence the contribution of the different cellular components involved in bone degradation. Similarly, in bone tumors from patients, different mechanisms may be active as a result of cancer heterogeneity and variation. In histological analyses of several osteosarcoma patient samples, we observed an apparent lack of osteoclasts in areas with cancer-induced bone degradation [70] while in another study, osteoclasts were identified in the tumors of 10 out of 16 patients, thus indicating patient-to-patient variation in the cellular distribution [84]. As an additional complicating factor, it is important to note that patient biopsies or tumor specimens from mouse cancer models, collected at single time points, are not representative of the entire course of disease. It is highly likely that the presence, distribution, and phenotype of different cell types change as the disease progresses.

**Figure 2:**
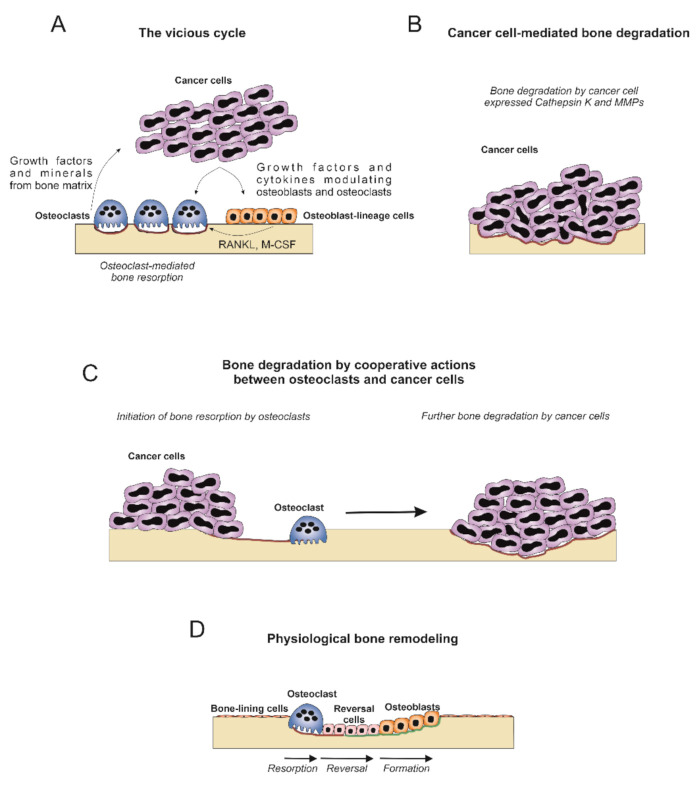
Three possible models of cancer-induced bone degradation. (**A**) In the vicious cycle model, cancer cells produce growth factors and cytokines that directly and indirectly stimulate exaggerated osteoclastogenesis and bone resorption. Growth factors and minerals released during osteoclast-mediated bone resorption further stimulate tumor growth. (**B**) In the model of cancer cell-mediated bone degradation, cancer cells are responsible for bone resorption through expression of collagenolytic proteases including Cathepsin K and MMPs. (**C**) Bone resorption is initiated by osteoclasts exposing eroded bone surfaces that attract cancer cells. These cancer cells will occupy zones of the eroded bone surface and contribute to further degradation through expression of collagenolytic enzymes. (**D**) Schematic representation of physiological bone remodeling. During the resorption phase, osteoclasts degrade the inorganic and organic bone component. Reversal cells in close contact with osteoclasts on eroded bone surfaces participate in degradation of the organic bone compartment and prepare the bone for the following formation phase. During the formation phase, osteoblasts deposit organic bone matrix that is subsequently mineralized.

Since the large majority of reports on cancer-related bone degradation assign the resorption capacity to the osteoclast (Figure 2A), the suggestion that tumor cells also have this capacity alone may seem controversial (Figure 2B). However, the observation of direct cancer-cell mediated osteolysis is not necessarily in contradiction with the predominant understanding of bone degradation and osteoclasts in cancer. Thus, we propose a model that involves a direct role of both osteoclasts and cancer cells in the bone degradation process (Figure 2C). This model takes into consideration the processes known from physiological bone remodeling (Figure 2D) and, in particular, the role of the reversal cell in this process. We propose that in some instances, the cancer cells take on a role that resembles the one of reversal cells during normal bone remodeling. A subset of reversal cells express the collagenase MMP13 and, interestingly, these cells appear to participate in the collagenolytic step of bone resorption through MMP-mediated actions [85,86,87,88].

Indications of a resemblance between reversal and cancer cells in this respect were obtained from histological analyses of human osteolytic osteosarcoma [70] and mouse cancer models with inoculation of melanoma [82] or myeloma cells [83] into the circulation, resulting in bone metastasis. In these studies, cancer cells were found to be in direct contact with the eroded bone, similar to the reversal cells in normal bone remodeling, which localize to zones of eroded bone and participate in osteoclast-initiated bone resorption [85]. Yet, in the normal physiological situation, reversal cells are observed to be in close contact with osteoclasts actively engaged in bone resorption [85], whereas only few osteoclasts were found in the vicinity of cancer-cell occupied bone surfaces [70,82,83]. However, the latter observation does not necessarily reflect that the osteoclasts were never present at these sites or that osteoclasts are not essential for bone degradation in these cases. We suggest that in these cases, even a low number of osteoclasts could be sufficient for initiation of osteolysis. Subsequently, cancer cells could occupy the eroded bone surfaces and extend the bone resorptive process. These pre-eroded bone surfaces may display destabilized hydroxyapatite crystals due to the preceding osteoclastic activity, thereby allowing the cancer cells to continue collagenolysis, as is the case for the reversal cells. In this context, it is relevant to note, that bone material used for in vitro studies of cancer cell osteolysis may resemble bone partially degraded by osteoclasts [70]. This may account for the apparent osteoclast-independent bone resorption by cancer cells in some in vitro studies.

**Figure 3:**
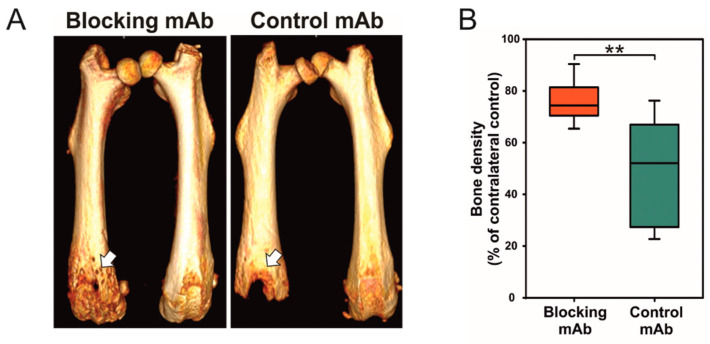
Functional targeting of the endocytic collagen receptor, uPARAP/Endo180, protects against bone destruction in an osteosarcoma mouse model (**A**) MicroCT scans of tumor and contralateral non-tumor bearing bones from mice treated with either an anti-uPARAP/Endo180 monoclonal antibody (blocking mAb) or an irrelevant control mAb. White arrow indicates the site of sarcoma cell inoculation. (**B**) Quantification of (**A**). The blocking mAb leads to a pronounced protection against tumor-dependent bone destruction. ** *p* = 0.002, Welch’s *t*-test. Reproduced with permission from Engelholm et al., Journal of Pathology; published by John Wiley and Sons, 2016 [70].

In a study of a syngeneic mouse model with intrafemoral osteolytic sarcoma growth, the functional targeting of the collagen receptor uPARAP/Endo180 led to a pronounced protection against bone destruction (Figure 3A,B) [70]. uPARAP/Endo180 is expressed by the sarcoma cells and bone cells of mesenchymal lineage while it is not expressed by the osteoclasts, indicating that the observed reduction in bone destruction was not caused by a direct osteoclast targeting. In physiological bone remodeling, uPARAP/Endo180 is strongly implicated in clearance of collagen fragments for intracellular degradation, while it is unlikely that it participates in the demineralization process necessary for bone resorption. Therefore, the above observation would fit well with a composite model (Figure 2C), in which the osteoclasts are necessary for initial osteolysis, while in some cancer types the tumor cells can also contribute to the ongoing process.

The interplay suggested above is not a uniform mechanism and the role of cancer cells is most likely highly variable. For most of the studies that imply a direct role of cancer cells in bone degradation, the strongest evidence is related to their collagenolytic activity. The expression of collagenases and gelatinases, including cathepsin K and various MMPs, in different types of cancer cells has been extensively studied [70,80,81,82,89,90,91,92,93,94]. However, it was recently shown that depletion of the collagenase MT1-MMP/MMP14 in osteolytic osteosarcoma had no effect on cancer-induced bone degradation in an orthotopic transplanted mouse model [94]. Thus, the mere expression of collagen-degrading proteases does not necessarily implicate that the cancer cells are osteolytic and the functional significance of cancer-cell-expressed collagenolytic enzymes is likely to differ between different types of cancers. Furthermore, to achieve ongoing bone resorption, demineralization through the creation of an acidic environment is a necessary step. Although some of the above-mentioned studies suggest that cancer cells are able to demineralize bone [79,81,82,83] and create resorption cavities [80,81,82], the underlying mechanisms have not been elucidated. In particular, the efficiency of the cancer cells’ contribution to this process as compared to that of osteoclasts has not been established.

Bone surfaces eroded by osteoclasts expose factors and extracellular matrix proteins that may attract the cancer cells to these areas. This is a mechanism that exists in physiological bone remodeling, where native collagen, exposed on eroded bone surfaces, has a haptotactic effect on cells of the osteoblast lineage [95]. It is indeed possible that similar haptotactic effects by exposed bone proteins could be relevant in some types of cancers. This may explain the observations of cancer cells engaged with eroded bone surfaces and provide a link between osteoclast-initiated bone resorption and cancer cell-mediated collagenolysis.

## 6. Modulatory Effects of Osteosarcoma Cells on Osteoclastogenesis

It is generally accepted that osteosarcoma modulates osteoclastogenesis. However, some conflicting observations exist on whether osteosarcoma causes an increase or a decrease in osteoclast formation and activity. Studies based on patient samples, cell culture model systems in vitro, or mouse models of primary bone cancer have in some cases come to different conclusions [5,44,49,63,84,96,97].

In a study using a human osteosarcoma cell line, it was shown that co-culturing of osteosarcoma cells with human peripheral blood mononuclear cells resulted in an increased number of osteoclasts (identified as being TRAcP-positive multinucleated cells) and an elevated resorptive activity [49]. Studies of mice with orthotopic inoculation of osteosarcoma cells have shown an increased number of osteoclasts in tumor-bearing compared to non-tumor bearing bone [44,63,96]. Additionally, in osteosarcoma patients, enzymatically active TRAcP 5b serum levels were found to be significantly reduced after chemotherapy and surgical removal of the tumor, compared to the time of diagnosis [84]. Together, these observations suggest that osteosarcoma cells possess osteoclast-modulating capacities that fit with the vicious cycle model.

However, there are also a few studies suggesting a negative effect of osteosarcoma on osteoclastogenesis [5,97]. Analyses of osteoclast numbers in biopsies from osteosarcoma patients, evaluated by TRAcP 5 mRNA levels and immunohistochemistry, revealed a reduction in osteoclast numbers compared to healthy controls. In addition, this reduction was more pronounced in the patients who developed metastases [5]. In the same study, a reduction in osteoclast numbers was also observed in mice with intrafemoral transplanted osteosarcoma cells compared to PBS treated mice. Several osteosarcoma cell lines were used in this study and, as for the patient samples, a more prominent reduction was observed in mice transplanted with metastatic compared to non-metastatic osteosarcoma cells lines [5].

In another study, the same group of authors analyzed gene expression in biopsies from chemo-naïve osteosarcoma patients [97]. Compared to non-malignant bone biopsies, osteosarcoma biopsies displayed a gene expression profile that was correlated with reduced osteoclastogenesis. Additionally, this profile was more prominent in patients with a poor response to chemotherapy [97]. A recent study also reported that a low number of osteoclasts in osteosarcoma bone biopsies was associated with a poor response to subsequent chemotherapy [98].

From all of these studies, it is clear that reciprocal regulatory mechanisms exist between osteosarcoma cells and osteoclasts, however, the outcome is at present less clear. As discussed by Endo-Munoz and co-authors [99], it is likely that the interplay between cancer cells and osteoclasts change during cancer progression. Since osteosarcomas are genetically unstable, novel mutations may rapidly accumulate, which could bring about changes in the modulatory effects exerted by the cancer cells on the osteoclasts. It is indeed possible that recording the changes in osteoclastogenesis throughout osteosarcoma establishment and progression could provide some nuance to the discussion.

## 7. Conclusions

Dysregulated bone remodeling, resulting in pathological bone degradation, is not only a devastating consequence of bone cancer, it also appears to be an essential component of bone cancer development. As discussed in this review, several decades of studies have revealed how cancer cells exploit and hijack physiological bone remodeling processes and have positioned the osteoclast as a central player in establishment and progression of bone tumors. However, suggestions that cancer cells may also possess a bone degrading capacity challenge the classical vicious cycle model. In this review, we have combined these observations with current knowledge on the physiological bone remodeling process into a new model of cancer-induced bone degradation. In this model, cancer cells adopt a role that may resemble that of the reversal cell and may allow both osteoclasts and cancer cells to participate in bone resorption. We hope that this model may inspire additional studies that could further clarify the contributions of and interplay between osteoclasts and cancer cells with respect to bone degradation. Moreover, there appears to be a need for further investigations of potential changes in osteoclast numbers and activity throughout the course of disease. We believe that such studies would considerably add to the current understanding of the contribution of osteoclasts in cancer-associated bone destruction and bone cancer progression in general.
